# Fluoroquinolone-resistant *Streptococcus pneumoniae*

**DOI:** 10.3201/eid1209.051400

**Published:** 2006-09

**Authors:** Mathias W.R. Pletz, Randolph V. Fugit, Lesley McGee, Jeffery J. Glasheen, Darcie L. Keller, Tobias Welte, Keith P. Klugman

**Affiliations:** * Emory University Rollins School of Public Health, Atlanta, Georgia, USA;; † Hannover Medical School, Hannover, Germany;; ‡ Denver Veterans Affairs Medical Center, Denver, Colorado, USA;; § University of Colorado Health Sciences Center, Denver, Colorado, USA;; ¶ University of Missouri-Kansas City School of Medicine, Kansas City, Missouri, USA;; # Emory University School of Medicine, Atlanta, Georgia, USA

**Keywords:** Streptococcus pneumoniae, Drug Resistance, Bacterial, Fluoroquinolones, Mutation, DNA Gyrase, Chronic Obstructive Pulmonary Disease, fitness, letter

**To the Editor:** In pneumococci, resistance to fluoroquinolones is associated with chromosomal mutations in the quinolone-resistance–determining regions (QRDR) of type II topoisomerase enzymes, predominantly *gyrA* and *parC*. Several mutations have been described in these enzymes, but only a few have been shown by in vitro studies to confer resistance: S81F or Y, C, or I and E85K in *gyrA*; E474K in *gyrB*; A63T, S79F or Y or L and D83G or N in *parC*; and E474K and D435N or H in *parE* (*1**–**5*). Other frequently described mutations are K137N in *parC* and I460V in *parE*, which appear to not contribute to fluoroquinolone resistance because they are commonly found in susceptible strains, and no evidence exists for their conferring fluoroquinolone resistance in vitro. We describe here a pneumococcal strain that was isolated from a 66-year-old white man with chronic obstructive pulmonary disease (COPD).

The patient was admitted to the hospital with a presumed exacerbation of COPD. He had been discharged from the hospital 2 days earlier, having recovered from a similar manifestation of this disease. His treatment history was 250 mg/day oral levofloxacin for 7 days while in the hospital and levofloxacin for 10 days as an outpatient for a similar lower respiratory tract infection 3 months earlier.

On this second admission he was given levofloxacin, 250 mg intravenously, once a day. He was treated with a low dosage because he was in renal failure. The patient continued to worsen and was transferred to the intensive care unit, where ceftriaxone, 1 g intravenously once a day, was given along with levofloxacin. He improved on the combination therapy and was discharged without sequelae.

Cultures of the patient's blood and sputum grew *Streptococcus pneumoniae*. The isolate from blood was resistant to levofloxacin (MIC 8 mg/L) and ciprofloxacin (MIC 8 mg/L), yet susceptible to gatifloxacin (MIC 1 mg/L) and ceftriaxone (MIC 0.38 mg/L), with intermediate resistance to penicillin (MIC 1.5 mg/L). The resistant isolate was of serotype 6A and of multilocus sequence type 376, which is the North Carolina^6A^-23 clone (http://www.sph.emory.edu/PMEN/index.html).

Efflux testing that compared the ciprofloxacin MICs in the presence and absence of reserpine (10mg/L) showed no evidence of an overexpressed efflux pump. We sequenced the QRDRs (*gyrA*, *gyrB*, *parC*, *parE*) and the entire *gyrA* and *parC* genes of the resistant strain isolated from blood by using previously described primers (*2*). Sequencing showed a S79Y mutation in *par*C and a Q118K (CAA→AAA) mutation in *gyr*A. Sequencing of the entire *gyrA* and *parC* genes confirmed that no additional amino acid substitutions were outside the QRDRs. The entire *gyrA* gene PCR product was transformed directly into the susceptible pneumococcal reference strain R6 by a standard transformation protocol (*4*). Transformants were selected on plates containing increasing concentrations of ciprofloxacin and, in a second step, were transformed with the entire *parC* gene of the resistant strain.

The ciprofloxacin and levofloxacin MICs of R6 transformed with the *gyrA* gene of the resistant isolate containing the new Q118K mutation were 4 and 2 mg/L, respectively. After additional transformation of these transformants with *parC* of the resistant isolate containing the S79Y mutation, the selected double transformants exhibited the same MICs as the original clinical isolate (8 mg/L for ciprofloxacin and levofloxacin). The transformation of *parC* alone conferred an intermediate increase in the MICs (ciprofloxacin 2 mg/L, levofloxacin 4 mg/L). All transformants were confirmed by sequencing.

To determine the biologic cost associated with the different resistance mutations in vitro, each fluoroquinolone-resistant mutant was competed against the fluoroquinolone-susceptible parent strain R6 (with an independent streptomycin resistance marker) as described by Johnson et al. (*6*). The outcome was evaluated as the change in the ratios of the competing strains as a function of the number of generations. Each competition was performed in triplicate by using independent starting cultures of each competing strain. Compared with the wild-type R6 strain, the relative fitness values for the *gyrA*, *parC*, and double mutants were 1.06, 1.03, and 0.93, respectively.

These data indicate that a single mutation in either *parC* or *gyrA* does not impose a substantial fitness burden. In contrast, the double-mutation *parC* S79Y and *gyrA* Q118K was associated with a slower growth rate. Similar results of relative fitness for single (*parC* S79Y and *gyrA* S81F) and double mutations were observed by Gillespie et al. (*7*).

Development of resistance to fluoroquinolones is a stepwise process, involving spontaneous mutations in the genes encoding the target enzymes DNA gyrase and the topoisomerase IV. Mutants with mutations in 1 of the enzymes are estimated to arise at a frequency of 1 to 10^-7^ (*1*). Therefore, fluoroquinolone resistance due to selection of spontaneous mutants during treatment may be related to the number of bacterial cells in the population under selective pressure. Patients with COPD are frequently colonized by high bacterial loads. COPD has been identified in several recent studies as an independent risk factor for fluoroquinolone resistance (*8**,**9*). Low doses of fluoroquinolones may also lead to an increased risk for resistance selection (*10*). Because the Q118K mutation has not been previously described, this new mutation was probably selected by the current or antecedent treatments rather than by an infection with a resistant widely disseminated clone.
